# Supplementary material for: 2040 greenhouse gas reduction targets and energy transitions in line with the EU Green Deal
Source: Nat Commun. 2026 Apr 16;17:3417. doi: 10.1038/s41467-026-71159-8 (PMC13087189; doi:10.1038/s41467-026-71159-8)
Supplement: Supplementary file 1 — Supplementary Information [file 41467_2026_71159_MOESM1_ESM.pdf]

Supplementary Information

2040 greenhouse gas reduction targets and energy transitions in line with the EU Green Deal

Renato Rodrigues[1], Robert Pietzcker[1], Joanna Sitarz[1,2], Anne Merfort[1,2], Robin Hasse[1,2], Johanna Hoppe[1,2], Michaja Pehl[1], Ahmad Murtaza Ershad[1], Jarusch Muessel[1,2], Felix Schreyer[1,2], Lavinia Baumstark[1], Gunnar Luderer[1,2]*

1- Potsdam Institute for Climate Impact Research, Potsdam, Germany

2- Global Energy Systems Analysis, Technische Universität Berlin, Berlin, Germany

*- Corresponding author: Renato Rodrigues - renato.rodrigues@pik-potsdam.de

SI1. Supplementary Discussion

We provide additional information on the following topics:

- Mitigation costs
- Regional results
- Sectoral energy use
- Greenhouse Gas (GHG) Emissions
- Carbon Dioxide Removal (CDR)
- VRE Integration

SI1.A Mitigation costs:

The implicit carbon prices required to meet the 2030 and 2050 targets under the European Green Deal are shown in Supplementary Fig. 1.:

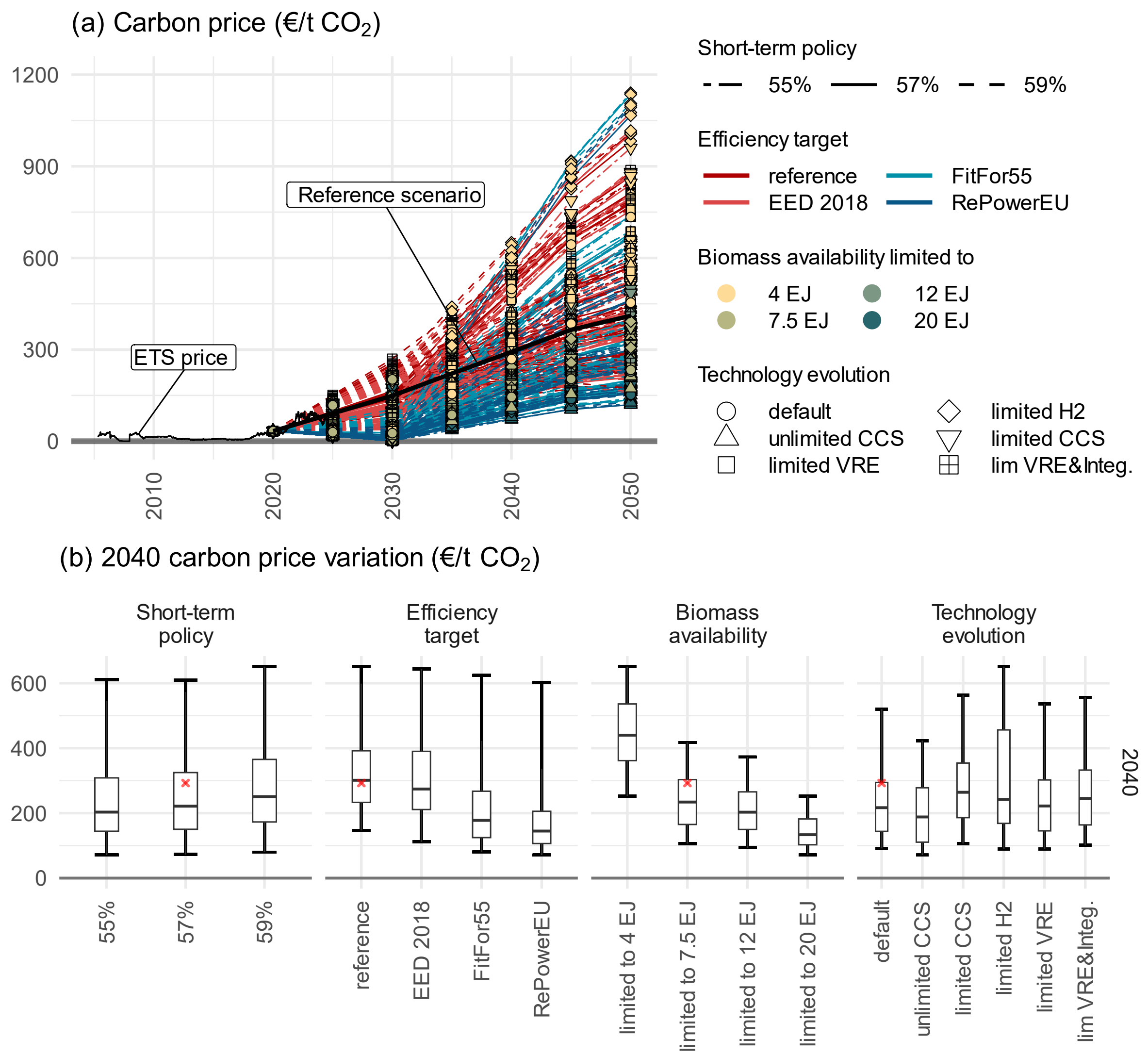

Supplementary figure 1. EU-27 carbon price. *Carbon price across all scenarios (a), and variation per sensitivity dimension (b). The black line in (a) and red cross symbols in (b) represent the results of the reference scenario. Box-plots in (b) show the range from first to third quartile of the sensitivity scenarios.*

Carbon price results are particularly sensitive to biomass availability (bioLim assumptions) and non-price-based mitigation policies, such as energy efficiency targets (e.g., EED 2018, Fit-for-55, RePowerEU). It is important to emphasise that the carbon prices presented here assume the continuation of a number of other existing policies, such as the CO2 emissions standards for cars and trucks. If some or all of the existing policies that accelerate the transformation were to be discontinued or weakened, carbon prices required to reach climate neutrality would increase further.

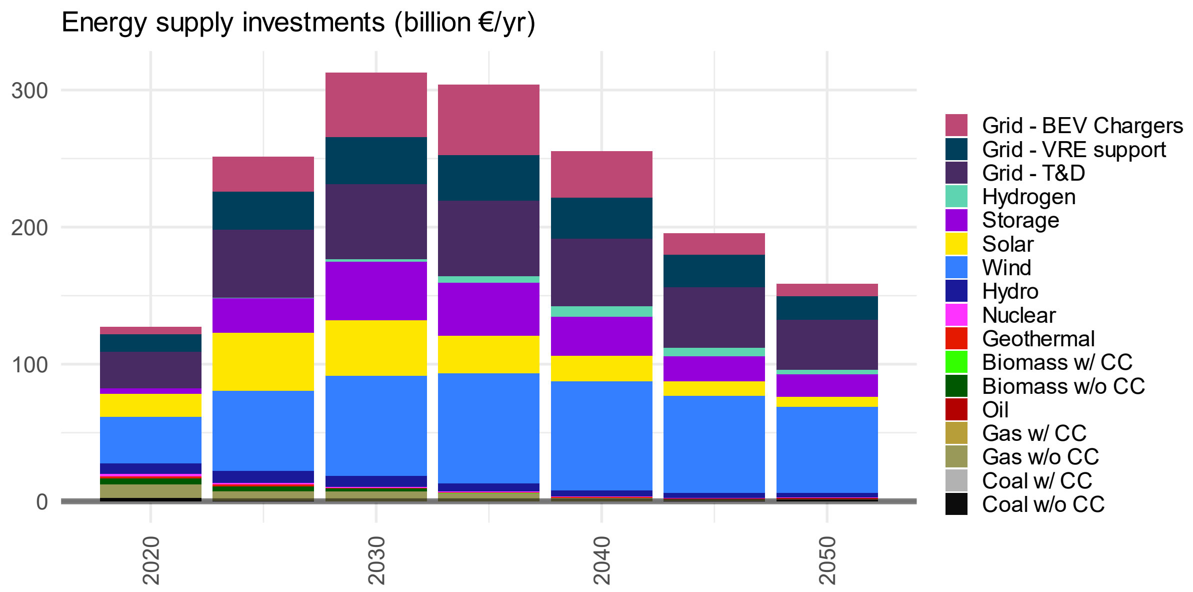

Supplementary figure 2. EU-27 mitigation costs*. Energy supply investments in the reference scenario.*

The majority of investment decisions to support the modernization of the energy supply and demand in the decarbonization process is centred around the 2030s decade (Supplementary Figure 2). Early implementation of financing mechanisms is critical to enable these investments.

SI1.B Regional results:

The simulations incorporate country-specific dynamics, including resource potentials (e.g., wind and solar), behavioral assumptions (e.g., transport modes), national policies (e.g., Germany's 2045 carbon neutrality target), and technology specific legislation and trends (e.g. coal phase-out and nuclear legislations).

While our analysis focuses on EU-27 results, key sectoral transformations remain consistent across individual countries. For instance, Germany and France both pursue rapid electrification and increased VRE deployment, supported by battery storage, to achieve power sector decarbonization (Supplementary Fig. 3 and Supplementary Fig. 4). Differences arise due to national policies, such as Germany's accelerated decarbonization and France's ongoing reliance on nuclear power. Future research could explore deeper country-level dynamics, including hydrogen market maturity and export opportunities for Iberian countries due to their favorable VRE potential.

Scenarios with higher variable renewables integration costs have a limited impact on installed capacity results, as these additional costs remain small relative to the overall system transformation.

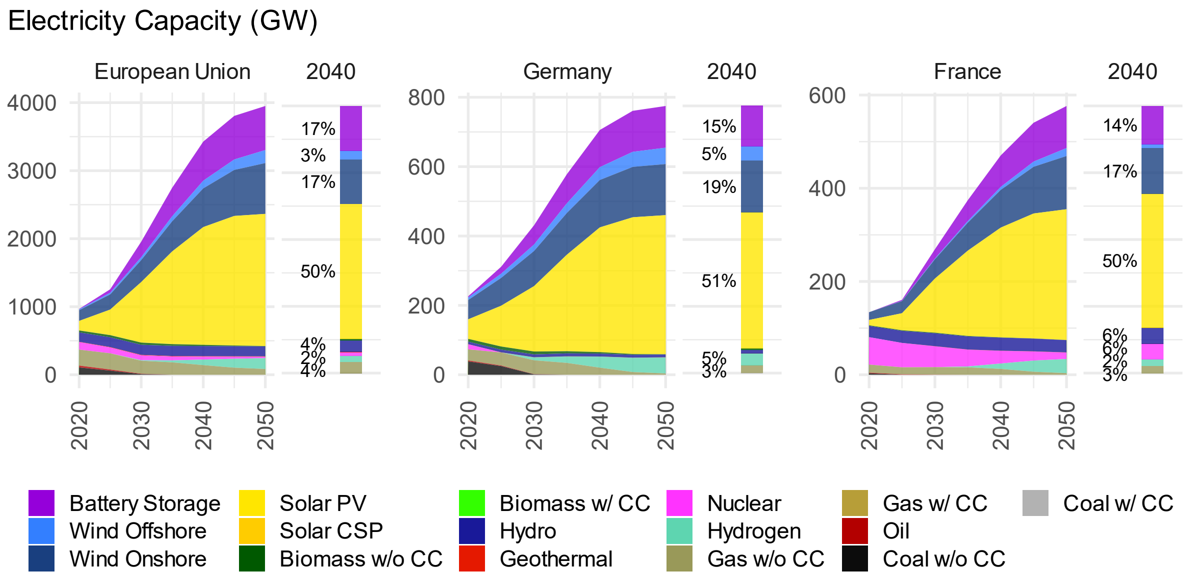

Supplementary figure 3. EU-27 Electricity capacity. EU-27, Germany, and France electricity capacity (GW) in the reference scenario.

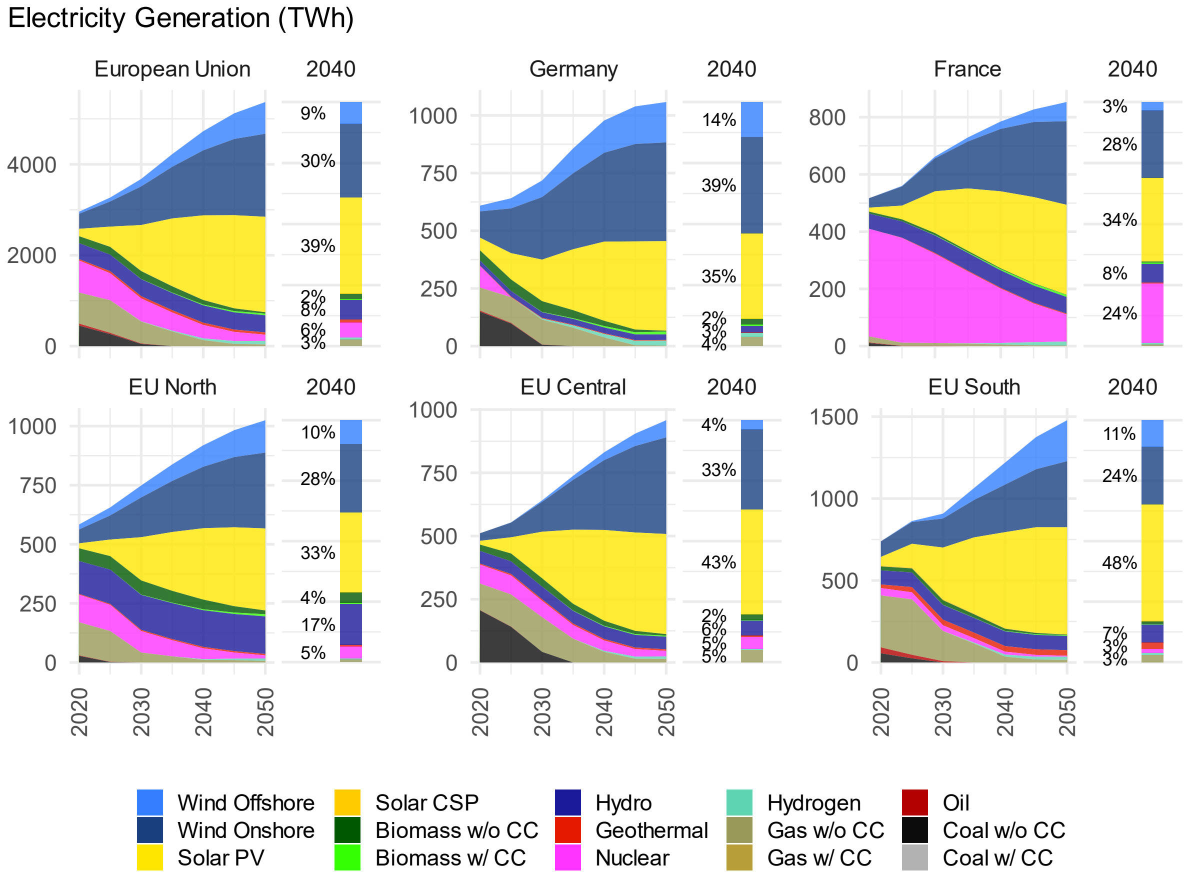

Supplementary figure 4. EU-27 Electricity generation. EU-27, Germany, France, north EU countries (EUN), central and east EU countries (EU Central), south and west EU countries (EU South), electricity generation (TWh) in the reference scenario.

SI1.C Sectoral energy use:

Electrification enhances energy efficiency, reducing total final energy use without necessarily compromising economic output (Supplementary Fig. 5a). However, residual fossil fuel use persists beyond 2040, primarily in industry and transport, necessitating emissions offsets to meet policy targets (Supplementary Fig. 5b).

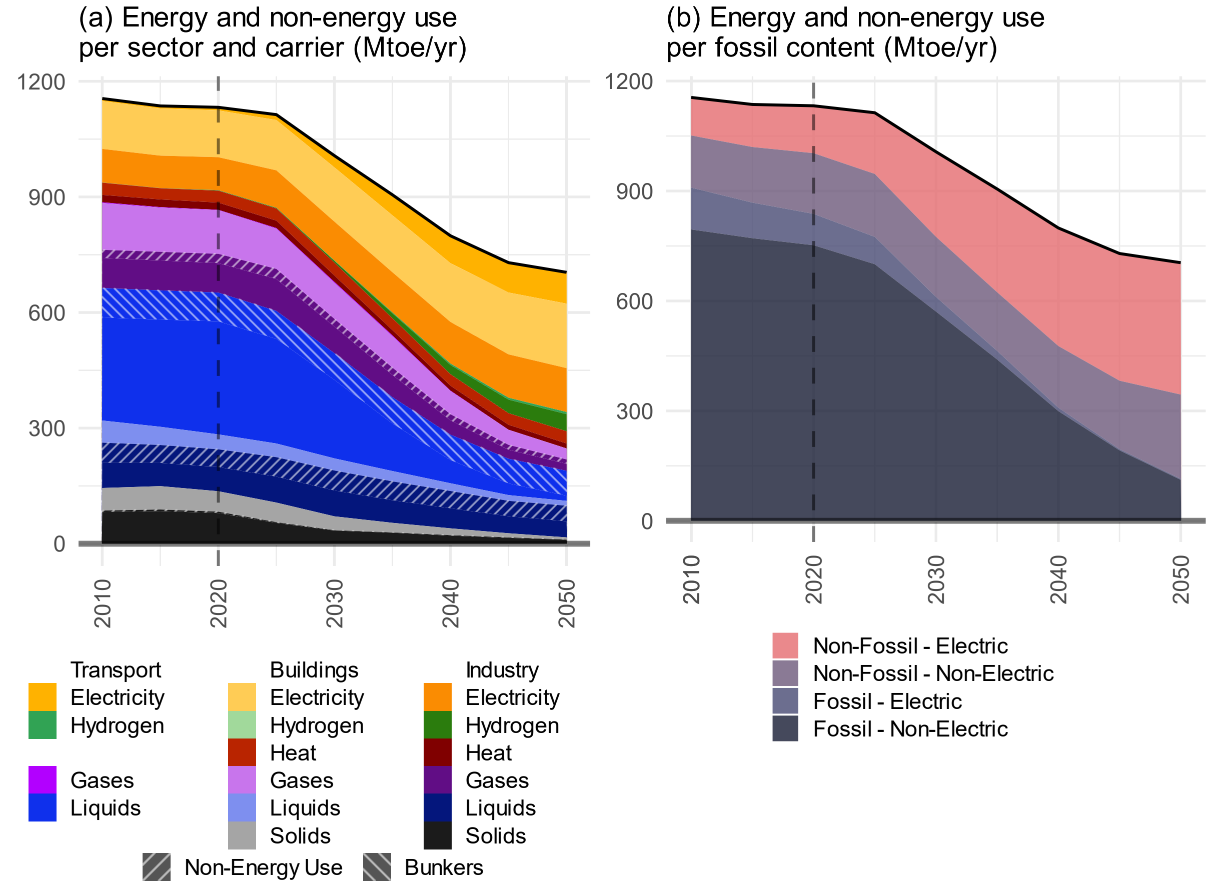

Supplementary figure 5. EU-27 Emissions. Energy and non-energy use in the reference scenario (a) per sector and carrier and (b) per fossil content.

SI1.D Greenhouse Gas (GHG) Emissions:

European anthropogenic carbon dioxide emissions decline rapidly by 2040 (Supplementary Fig. 6), but methane emissions, particularly from agriculture and waste, require additional regulatory measures beyond the scope of our scenarios.

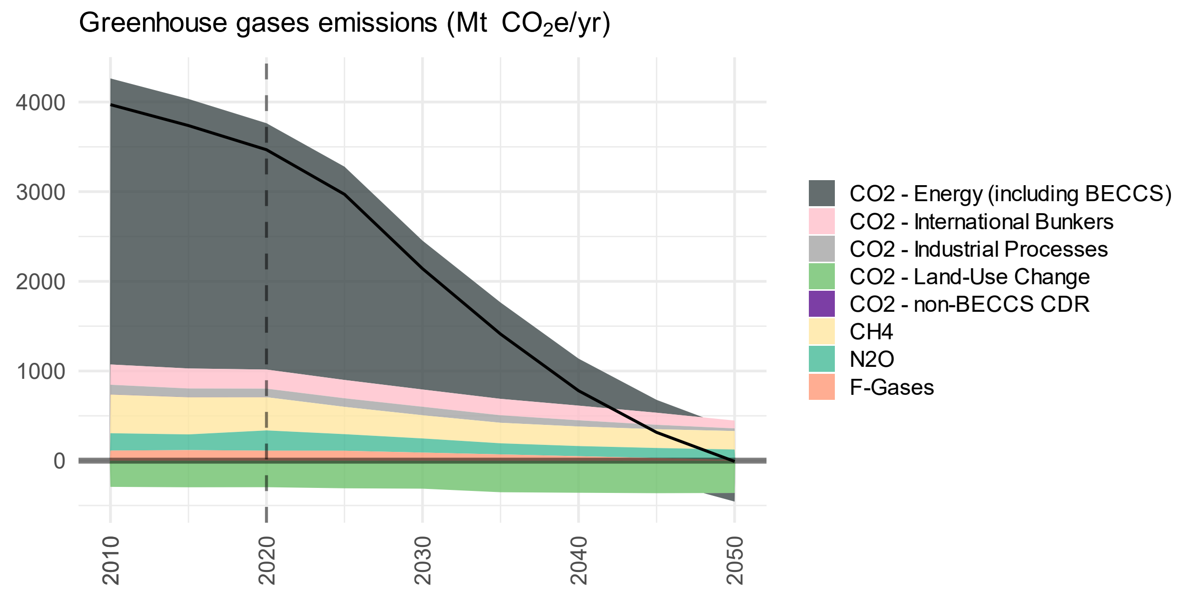

Supplementary figure 6. EU-27 GHG gases. Emissions by gas in the reference scenario (CO_2eq).

Agriculture remains a critical challenge due to its political and institutional rigidity and the absence of targeted mitigation policies. Addressing agricultural emissions will require further research and policy innovation.

SI1.E Carbon Dioxide Removal (CDR):

Bioenergy with Carbon Capture and Storage (BECCS) is the primary CDR option in our scenarios, reaching 131 MtCO2/yr (sensitivity range: 44 MtCO2/yr to 191 MtCO2/yr) by 2040 (Supplementary Fig. 7a). However, BECCS deployment is limited by biomass availability. Under stringent biomass constraints (bioLim 4), BECCS capture can be reduced to as lower as 43 MtCO2/yr by 2040, increasing the need for alternative emission compensation technologies such as Direct Air Capture (DAC), which may reach up to 19 MtCO2/yr by 2040 in scenarios with limited hydrogen and e-fuels availability. By contrast, variations in geological CO_2 injection capacity have a much stronger effect on the carbon price levels required to incentivize deployment, while having only a limited impact on overall CDR volumes.

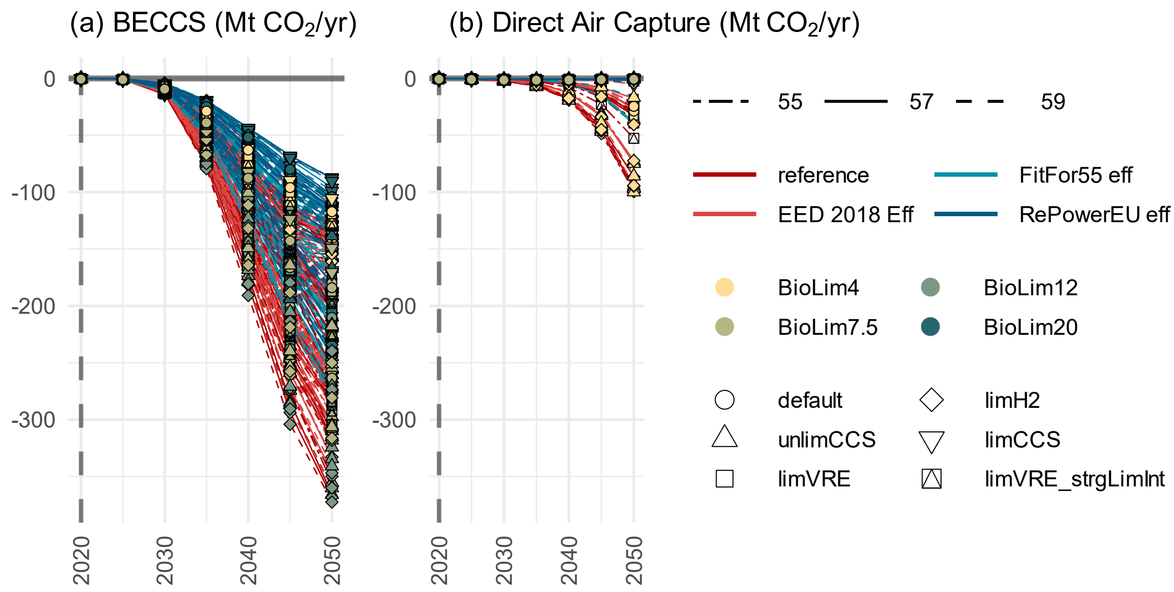

Supplementary figure 7. EU-27 CDR. CDR deployment: (a) BECCS and (b) DACCS.

SI1.F VRE integration:

Transforming a power sector based to mostly thermal dispatchable power plants to one based mostly on wind&solar requires substantial effort and is accomplished through a combination of grid expansion for interregional pooling, battery storage for balancing short-term mismatches between supply and demand, flexibilized energy demands and hydrogen generation, and curtailment of surplus VRE generation. The REMIND model captures the challenges and options related to the temporal and spatial variability of wind and solar power in aggregated ways[1]. Generally, IAMs are too aggregated to give very detailed information about the exact setup of future electricity systems, such as time-resolved dispatching and curtailment levels, spatial layout of the grid and power plant positioning, costs for ramping and redispatch. However, it is possible to develop aggregated representations of integration challenges of variable renewable energies in IAMs that lead to aggregated results (technology deployment, generation mix, average prices) that are consistent with the ranges produced by detailed, hourly-resolved and spatially explicit investment-and-dispatch models under similar scenario settings.

Building on theoretical analyses of the economic and technical integration challenges for wind and solar[2,3,4], we developed and refined approaches to represent the most relevant aggregated VRE integration effects in the large-scale IAM REMIND[1,5,6]. A validation study showed that when comparing scenarios spanning a range of wind and solar shares in electricity generation, the aggregated results from REMIND agree reasonably well with the results from the hourly-resolved detailed investment-and-dispatch model REMIX[7].

SI2. Supplementary Notes

Our results account for nationally originated emissions and international transport bunker emissions. Future research could include embedded carbon in imports and policies, such as carbon border adjustments, to assess the decarbonization potential of traded goods.

All monetary figures are presented in euros, adjusted to 2025 price levels.

Below you can find additional details to improve understanding and reproducibility of these paper results:

- Figure 1:
  - Figure 1a: Total GHG emissions include LULUCF and international transport.
  - Figure 1b: Emission reductions relative to 1990 emissions include LULUCF and intra-EU aviation only (4713 Mt CO_2e), except for 2045 and 2050 emission reductions which are calculated relative to 1990 emissions including LULUCF and international transport (4814 Mt CO_2e). A 60% emission reduction by 2030 can result from a specific combination of sensitivity parameters — particularly under higher energy efficiency assumptions (as in the RePowerEU efficiency scenarios) — leading to a slight overachievement in some scenarios targeting a 59% reduction.
  - Figure 1d: whiskers denote the full scenario range (0th to 100th percentile). Percentage reductions are relative to the 2018-2022 average emission values.
- Figure 5:
  - Chart symbols:
    - Dashed lines: historical values.
    - Pointed lines: policy targets or EU goals.
    - Box plots: interquartile range and median (first quartile, 25th percentile, median, 50th percentile, and third quartile, 75th percentile, of all scenarios results.
    - Whiskers: full range of all scenario results (0th to 100th percentile).
    - Shaded areas: scenario distribution (violin plots).
    - Red crosses: reference scenario results.
  - Indicator details:
    - Emissions reductions: Relative to 1990 levels, including LULUCF and intra-EU aviation only.
    - Energy demand: Final energy consumption excluding non-energy use and international bunkers.
    - Renewables share in final energy: Includes geothermal, hydro, onshore and offshore wind, solar, biomass (heat, solids), solar heat, hydrogen from biomass, biogases, bioliquids, and other bioenergy sources.
    - Residual fossil emissions: Includes emissions from international bunkers and industrial processes.
    - Electricity demand: Final electricity consumption, excluding electricity used by electrolyzers and large-scale heat pumps supplying district heating.

Supplementary References

1. Pietzcker, R., Stetter, D., Manger, S., Luderer, G. Using the sun to decarbonize the power sector: The economic potential of photovoltaics and concentrating solar power. Applied Energy, Volume 135, Pages 704-720, ISSN 0306-2619 (2014). https://doi.org/10.1016/j.apenergy.2014.08.011
2. Ueckerdt, F., Hirth, L., Luderer, G., Edenhofer, O. System LCOE: What are the costs of variable renewables? Energy, Volume 63, Pages 61-75, ISSN 0360-5442. (2013) https://doi.org/10.1016/j.energy.2013.10.072.
3. Hirth, L., Ueckerdt, F. Edenhofer, O. Integration costs revisited – An economic framework for wind and solar variability. Renewable Energy, Volume 74, Pages 925-939, ISSN 0960-1481. (2015) https://doi.org/10.1016/j.renene.2014.08.065.
4. Ueckerdt, F., Brecha, R., Luderer, G. Analyzing major challenges of wind and solar variability in power systems Renewable Energy, Volume 81, Pages 1-10, ISSN 0960-1481. (2015) https://doi.org/10.1016/j.renene.2015.03.002.
5. Ueckerdt, F., Brecha, R., Luderer, G., Sullivan, P., Schmid, E., Bauer, N., Böttger, D., Pietzcker, R. Representing power sector variability and the integration of variable renewables in long-term energy-economy models using residual load duration curves, Energy, Volume 90, Part 2, Pages 1799-1814, ISSN 0360-5442. (2015) https://doi.org/10.1016/j.energy.2015.07.006.
6. Ueckerdt, F., Pietzcker, R., Scholz, Y., Stetter, D., Giannousakis, A., Luderer, G. Decarbonizing global power supply under region-specific consideration of challenges and options of integrating variable renewables in the REMIND model. Energy Economics, Volume 64, Pages 665-684, ISSN 0140-9883. (2017) https://doi.org/10.1016/j.eneco.2016.05.012.
7. Pietzcker, R., Ueckerdt, F., Carrara, S., Sytze de Boer, H., Després, J., Fujimori, S., Johnson, N., Kitous, A., Scholz, Y., Sullivan, P., Luderer, G. System integration of wind and solar power in integrated assessment models: A cross-model evaluation of new approaches. Energy Economics, Volume 64, Pages 583-599, ISSN 0140-9883. (2017) https://doi.org/10.1016/j.eneco.2016.11.018.
